# Correction: Predictive ability of plasma concentration of triglyceride/high density lipoprotein-cholesterol ratio for cardiometabolic variables in a sub-Sahara black African adolescent population–Nigerians

**DOI:** 10.1371/journal.pone.0347509

**Published:** 2026-04-16

**Authors:** The PLOS One Staff

## Notice of Republication

This article was republished on March 23, 2026, to correct errors with the figures. Figures 2,3 and 4 were omitted in the original article. The publisher apologizes for the errors. Please download this article again to view the correct version. The originally published, uncorrected article and the republished, corrected articles are provided here for reference.

## Supporting information

S1 File Originally published, uncorrected article. (PDF)

S2 File Republished, corrected article. (PDF)
