# Supplementary material for: Predictive ability of plasma concentration of triglyceride/high density lipoprotein-cholesterol ratio for cardiometabolic variables in a sub-Sahara black African adolescent population–Nigerians
Source: PLoS One. 2025 Aug 18;20(8):e0322266. doi: 10.1371/journal.pone.0322266 (PMC12360606; doi:10.1371/journal.pone.0322266)
Supplement: Attachment — Submitted filename: report_10.1101+2025.03.21.25324366.pdf [file pone.0322266.s001.pdf]

ScreenIT Report

This is an automated report for 10.1101+2025.03.21.25324366.pdf. This report was solicited by the PLOS One editorial team and provided by ScreenIT.

ScreenIT is an independent group of scientists developing automated tools that analyze academic papers. A set of automated tools screened your submitted manuscript and provided the report below. Each tool was created by your academic colleagues with the goal of helping authors.

The tools look for factors that are important for transparency, rigor and reproducibility, and we hope that the report might help you to improve reporting in your manuscript. Within the report you will find links to more information about the items that the tools check. These links include helpful papers, websites, or videos that explain why the item is important.

While our screening tools aim to improve and maintain quality standards they may, on occasion, miss nuances specific to your study type or flag something incorrectly. Each tool has limitations that are described on the ScreenIT website. The tools screen the main file for the paper; they are not able to screen supplements stored in separate files.

Please note that the Academic Editor had access to these comments while making a decision on your manuscript. The Academic Editor may ask that issues flagged in this report be addressed.

If you would like to provide feedback on the ScreenIT tool, please email the team at ScreenIt@bih-charite.de. If you have questions or concerns about the review process, please contact the PLOS One office at plosone@plos.org.

How to read the report:

- no action required

- review for potential issues

- a critical issue was found by a tool

- may not be applicable to your paper

- more information about this item can be obtained using this link

Rigor

✓	Ethics	Field Sample Permit: *"The study, descriptive and cross-sectional, was conducted at Biochemistry and Nutrition Department of the Nigerian Institute of Medical Research (NIMR), Lagos, Nigeria."*<br>IRB: *"On the 4th of February 2019, the Institutional Review Board of the Nigerian Institute of Medical Research (NIMR IRB (IRB/ 18/062) approved the study protocol, and the study was carried out in accordance with the Declaration of Helsinki (2000)."*<br>Consent: *"Thereafter, students who, in the presence of their teachers, gave verbal assent to participate in the study, were then given consent forms for their parents to also read, understand and sign or thumbprint."*
✓	Inclusion and Exclusion Criteria	*"Eligibility criteria: To be eligible, participants must be 10-19 years of age, dark-skinned and indigenous citizen of Nigeria. He or she must be resident in the community for a minimum of two years in the respective area of the study, must also be a regular student at any of the selected secondary schools."*
?	Flow charts and attrition	We did not find a study flow chart of excluded observations. We strongly recommend using flow charts because they provide an overview of the study design and more information about attrition. If you included a study flow chart in your supplemental files, we apologize for missing this. Our tool is not able to screen separate supplemental files.<br><br>Sentence about attrition: not detected. Please provide information about the drop-out of subjects, or loss of animals or samples. This could be done using a study flow chart, or described in the text.
✓	Sex as a biological variable	*"Also excluded were females who were (i) pregnant (ii) suspected to be pregnant (iii) breastfeeding and (vi) those on oral contraceptives."*

✓	Subject Demographics	Age: *"In the study, eligible adolescents aged 10-19 years, were selected from different secondary schools for a dietary and nutrition survey (DNS) and primary data were collected from them."*
?	Randomization	Not detected. If you performed an experimental study, please specify whether participants, animals or samples were randomly assigned to treatments or groups, and specify the randomization procedure.
?	Blinding	Not detected. Please specify whether blinding was used at various phases of the experiment (e.g., blinding of patients, caregivers, outcome assessments, data analysis).
✓	Power Analysis	*"Sample size: The sample size, sampling technique and procedure for the study have also been reported in an earlier publication [28]."*
n/a	Replication	not required.

Generated by Barzooka (flow charts), SciScore (everything else).
SciScore only screens the text of the methods section; we apologize if the tool has missed items reported elsewhere in the paper or supplements.

Resources

No key resources detected.
Generated by SciScore. SciScore only screens the text of the methods section; we apologize if the tool has missed items reported elsewhere in the paper or supplements.

Transparency

?	Open code	If you wrote code to analyze your data, please consider sharing the code in a public repository. This makes it easier for others to reproduce your analyses, and may also aid others seeking to analyze similar datasets.
?	Open data	If permitted, sharing data on a public repository with a persistent identifier (a DOI or accession number) can improve reproducibility and make it easier for other scientists to expand on your work. Papers with open data are cited more often than papers without open data. Some institutions have an expert who can provide advice on data sharing.

✓	Self-acknowledged limitations	*"Strengths and limitations However, the study has some limitations that need further consideration. First, the sample size is relatively small. A larger sample size would probably have given a more robust analysis and extend its external validity. Second, bias may have been introduced in the sample selection as the proportion of girls outweighed that of boys, and some of the overweight or obese students may have been left out. Thirdly, the study was conducted in the southwest corner of Nigeria, on the Atlantic Ocean coastline thus it may not be representative of Nigerian adolescent population in the arid eco-system, the population living in high altitude region in north-central part of the country or those resident in the rain-forest zone. Fourthly, this was a cross-sectional study thus devoid of predicting any prognosis nor does it assume cause and effect of any findings. Finally, the study did not consider anthropometric status such as waist circumference, Body Mass index-for-age to d..."*
✓	Conflict of interest statement	Thank you for stating any conflicts of interest.
✓	Funding statement	Thank you for reporting your funding source(s), or stating that there was no specific funding for this work.
n/a	Registration statement	A registered protocol makes the planned research project more transparent and can protect against bias. Please consider registering future confirmatory studies.

Generated by ODDPub, limitation-recognizer, rtransparent, trial-identifier.

Figures

✓	No bar graphs of continuous data	Thank you for not using bar graphs to present continuous data. Many different datasets and data distributions can lead to the same bar graph, and the actual data may suggest different conclusions from the summary statistics alone.
✓	No rainbow color maps	Thank you for not using rainbow colormaps. Rainbow color maps distort readers' perception of the data by introducing visual artifacts and are not accessible to colorblind readers.

Generated by JetFighter, Barzooka.
