# Supplementary material for: Predictive ability of plasma concentration of triglyceride/high density lipoprotein-cholesterol ratio for cardiometabolic variables in a sub-Sahara black African adolescent population–Nigerians
Source: PLoS One. 2025 Aug 18;20(8):e0322266. doi: 10.1371/journal.pone.0322266 (PMC12360606; doi:10.1371/journal.pone.0322266)
Supplement: Attachment — Submitted filename: The Editor PLOS-ONE.docx [file pone.0322266.s003.docx]

The Editor-in-Chief,

My collegue and I would like to express our gratitute to you and the two reviewers who had gone through our original manuscript titled “Predictive Ability Of Plasma Concentration Of Triglyceride/High Density Lipoprotein-Cholesterol Ratio For Cardiometabolic variables In A Sub-Sahara Black African Adolescent Population – Nigerians.” We appreciate the efforts of everyone to make this manuscript as welcoming and enjoyable to read, as much as possible. We have followed the Editor’s remarks one by one and responded to them. All the remarks and corrections suggested by the reviewers have been effected.

Rebutal

However, we would like to crave your indulgence to defer the inclusion of TgG ratio to a future paper. The main reason for this deferment is that there are already, too many information included in the current manuscript and we would like to avoid readers’ fatigue. Another reason is that, including TgG ratio will cause a complete overall of the current paper. For these reasons, we wish to kindly say that we would consider a separate manuscript in the future to evaluate TgG ratio as a valid instrument of determining metabolic syndrome among Nigerian adolescents.

We are also happy to inform your good offices that we have included a flowchart into the paper as suggested.

Response to Editor and Reviewers

We hereby confirm that our submission contains all raw data required to replicate the results of our study.

Reviewer 1’s comment

1. *In the Introduction part, they should include a more detailed discussion regarding the attributes and clinical usefulness of the TG/HDL ratio. A pertinent review article referring to the subject is: Kosmas CE, Rodriguez Polanco S, Bousvarou MD, Papakonstantinou EJ, Peña Genao E, Guzman E, Kostara CE. The Triglyceride/High-Density Lipoprotein Cholesterol (TG/HDL-C) Ratio as a Risk Marker for Metabolic Syndrome and Cardiovascular Disease. Diagnostics (Basel). 2023 Mar 1;13(5):929. doi: 10.3390/diagnostics13050929. PMID: 36900073; PMCID: PMC10001260.I feel that a brief discussion of the above review article and its addition to the references would be useful.*

Our response: We have included and highlighted a brief discussion regarding the attributes and clinical usefulness of the TG/HDL-C ratio in the Introduction segment of the manuscript.

Reviewer 2’s comments using ScreenIT software:

**Flow charts and attrition**

*We did not find a study flow chart of excluded observations. We strongly recommend using flow charts because they provide an overview of the study design and more information about attrition. If you included a study flow chart in your supplemental files, we apologize for missing this. Our tool is not able to screen separate supplemental files.*

*Sentence about attrition: not detected. Please provide information about the drop-out of subjects, or loss of animals or samples. This could be done using a study flow chart, or described in the text.*

Our response: Flow chart and attrition have been included in the manuscript.

**Randomization**

*Not detected. If you performed an experimental study, please specify whether participants, animals or samples were randomly assigned to treatments or groups, and specify the randomization procedure.*

Our response: We did not perform any experimental study.

**Blinding**

*Not detected. Please specify whether blinding was used at various phases of the experiment (e.g., blinding of patients, caregivers, outcome assessments, data analysis).*

Our response: Blinding was not used in any stage of the study. The study was not experimental.

**Transparency**

*If you wrote code to analyze your data, please consider sharing the code in a public repository. This makes it easier for others to reproduce your analyses, and may also aid others seeking to analyze similar datasets.*

*If permitted, sharing data on a public repository with a persistent identifier (a DOI or accession number) can improve reproducibility and make it easier for other scientists to expand on your work. Papers with open data are cited more often than papers without open data. Some institutions have an expert who can provide advice on data sharing.*

Response: We did not use code in our data analysis.
