# Supplementary material for: Correction: Predictive ability of plasma concentration of triglyceride/high density lipoprotein-cholesterol ratio for cardiometabolic variables in a sub-Sahara black African adolescent population–Nigerians
Source: PLoS One. 2026 Apr 16;21(4):e0347509. doi: 10.1371/journal.pone.0347509 (PMC13086320; doi:10.1371/journal.pone.0347509)
Supplement: S1 File — (PDF) [file pone.0347509.s001.pdf]

PLOS One

RESEARCH ARTICLE

Predictive ability of plasma concentration of triglyceride/high density lipoprotein-cholesterol ratio for cardiometabolic variables in a sub-Sahara black African adolescent population–Nigerians

Susan J. Holdbrooke[1‡], Bamgboye M. Afolabi[1,2‡*]

1 Department of Biochemistry and Nutrition, Nigerian Institute of Medical Research, Lagos, Nigeria,
2 Health, Environment and Development Foundation, Lagos, Nigeria

‡ SJH and BMA are joint first authors.
* bmafolabi@gmail.com

Abstract

Introduction

This study aimed to determine whether triglyceride/high-density lipoprotein cholesterol (TG/HDL-c) ratio, which has been shown to be an innovative proxy marker of atherogenic indicator in the human plasma, and an indicator of the metabolic syndrome (MetS) and insulin resistance (IR), can predict systolic hypertension (SHT), diastolic hypertension (DHT), diabetic fasting plasma glucose (dFPG), hypertrigly ceridemia (HTG), hypercholesterolemia (HCHOL), low high-density lipoprotein-cholesterol (HDL-c) and high low-density cholesterol (LDL-c) in the Nigerian adolescent population living in metropolitan Lagos, Southwest Nigeria.

Methods

A dietary and nutritional survey (DNS) was conducted from October ~~2007~~ to March ~~2010~~. A total of 650 adolescent participants were recruited using a four-stage stratified sampling method but statistical analysis was restricted to the 613 who had complete anthropometric and clinical data. The sensitivity, specificity, and distance to the corner on the receiver operating characteristic (ROC) curve in each TG/HDL level were calculated. The shortest distance in the ROC curves was used to determine the optimal cutoff of the TG/HDL-c ratio for detecting MetS, Systolic and diastolic hypertension and diabetes.

Results

In all the subjects, the median TG/HDL-c ratio was significantly higher (P-value <000001) only among those with, than those without MetS (8.2 vs 3.0), those with

OPEN ACCESS

Citation: Holdbrooke SJ, Afolabi BM (2025) Predictive ability of plasma concentration of triglyceride/high density lipoprotein-cholesterol ratio for cardiometabolic variables in a sub-Sahara black African adolescent population–Nigerians. PLoS One 20(8): e0322266. https://doi.org/10.1371/journal.pone.0322266

Editor: Paolo Magni, Università degli Studi di Milano, ITALY

Received: March 19, 2025

Accepted: June 17, 2025

Published: August 18, 2025

Peer Review History: PLOS recognizes the benefits of transparency in the peer review process; therefore, we enable the publication of all of the content of peer review and author responses alongside final, published articles. The editorial history of this article is available here: https://doi.org/10.1371/journal.pone.0322266

Copyright: © 2025 Holdbrooke, Afolabi. This is an open access article distributed under the terms of the Creative Commons Attribution License, which permits unrestricted use,

distribution, and reproduction in any medium, provided the original author and source are credited.

Data availability statement: All relevant data and code have been uploaded to Figshare at DOI https://doi.org/10.6084/m9.figshare.29618150.

Funding: The author(s) received no specific funding for this work.

Competing interests: The authors have declared that no competing interests exist.

and without hypertriglyceridemia (4.2 vs 1.3), hypercholesterolemia (5.3 vs 2.8), low high-density lipoprotein-cholesterol (7.2 vs 2.5) and high low-density lipoprotein-cholesterol (3.3 vs 1.8, P-value = 0.0001). The prevalence of systolic hypertension, diabetic FPG, total cholesterol and LDL-c was higher with higher TG/HDL-c ratio while that of HDL-c was lower with higher TG/HDL-c in all subjects. TG/HDL-c ratio was strong in predicting dyslipidemia, especially hypertriglyceridemia and hypercholesterolemia in early and mid-adolescents and cardiometabolic risk factors of two or more in adolescents. An inverse relationship was observed between the stages of adolescence and cutoffs for MetS.

Conclusions

TG/HDL-c ratio effectively predicted MetS, hypertriglyceridemia and hypercholesterolemia among early and mid-adolescent Nigerians. As a tool, the TG/HDL-c ratio should be considered for the initial prediction of MetS and some of its dyslipidimic components. Further studies are needed to confirm findings in this study.

Introduction

The aging progression and metabolism in humans are intimately coupled, and various age-related alterations in body components, such as additional central adiposity and sarcopenia, have their foundations in fundamental aging processes, further exacerbated by a sedentary lifestyle [1]. Dysregulated metabolism has been identified as one of the hallmarks of aging mechanisms [2,3]. A study remarks that, due to the aging process, the elderly are at a higher risk of developing intra-abdominal obesity and metabolic syndrome [4]. A study reported the prevalence of MetS in an elderly population as varying from 11% to 43% (median 21%) according to the WHO, and 23% to 55% (median 31%) according to NCEP. Another study pronounced that MetS in the elderly is a verified risk factor for cardiovascular (CV) morbidity, especially stroke and coronary heart disease (CHD), and mortality [5] But what is the picture in pre-adulthood period of life, especially in adolescence and most especially among a Black African adolescent population? There is a consensus that MetS consists of obesity, insulin resistance, dyslipidemia, and hypertension [6,7] leading to increased risk of cardiovascular disease (CVD) and renal events [8]. The foremost cause of morbidity and mortality that impose substantial social and economic burden globally, are cardiovascular diseases (CVDs) [9]. Tong et al concluded that the burden of CVD in adolescent and young adults is a significant global health challenge [10]. Furthermore, Sun et al submitted that the countries/territories with low and low-middle SDI had a higher burden of CVDs than the countries/territories with high and high-middle socio-demographic index and concluded that there was (and probably still is) a substantial global burden of CVDs in youths and young adults in 2019 [9]. A systematic review and meta-analysis shows an upward trend in the population-based prevalence of CHD in Africa [11]. In urban Nigeria, there has been a rising prevalence

of hypertension [12]. A 150% increase in the prevalence of cardiovascular disease has also been reported [13]. Hypertension affects up to 46% of Nigerian adults and a rising proportion of Nigerian adolescents [14,15]. Major cooking oils in Nigeria are palm oil and groundnut oil, which are locally produced and sold at markets. Because these low-cost oils are widely available, people consume them regularly, which contributes to the rise in obesity, metabolic syndrome, and type 2 diabetes [16,17] thus associating food consumption to the development of MetS and invariably CVD. A study has reported dietary intake among adolescents with MetS, with the conclusion that high weekly consumption of animal protein appears to be a risk factor for the development of MetS, especially among boys [18]. These is ample evidence to suggest that serum lipid levels are rising in Nigeria. For example, in 2010, one study reported that the lipid profiles of adolescent Nigerians were low but gradually rising towards values seen in places where coronary heart disease occurs in epidemic proportions, indicating an increased risk of arteriosclerosis in future generations [19]. Later in 2015, Yar'adua et al reported a low risk for cardiovascular disease among Nigerian adolescents [20]. However, in 2021, Onyiriuka et al reported dyslipidaemia in a high proportion of adolescents with either overweight/obesity or normal BMI, with the former having a higher proportion and that low HDL-c level was the most common abnormality in dyslipidemia [21]. Although the majority of clinical CVD events occur at middle and older ages, substantial evidence indicates that atherosclerosis has its origins in childhood [22]. Over a century ago, Klotz and Manning [23] in autopsy findings, reported fatty streaks in large arteries of 6-year-old children. Of recent, intimal lesions were also observed in all aortas and in more than half of the right coronary arteries of adolescents aged 15–19 years [24]. Thus, without effective intervention in adolescence, atherosclerosis-related diseases may soar in the near future in Nigeria because of abnormal lipid metabolism which has been confirmed as an integral aspect of atherosclerotic pathogenesis, essential to the occurrence of CVD. Initially proposed by Gaziano et al, TG/HDL-c ratio refers to an atherogenic index which has been confirmed as a major independent predictor of cardiovascular disease [25]. Kannel et al [26] and Bertoluci et al [27] associated a high TG/HDL-c ratio to the risk of cardiovascular events. TG/HDL-C ratio has been shown to be a more dependable substitue for forecasting MetS and cardiometabolic risks compared to other lipid ratios such as TC/HDL-C, LDL-C/HDL-C and non-HDL-C/HDL-C with a high predictive value [28–30]. Ample examples exist of elevated cardiometabolic risk for TG/HDL-C ratios between 2 and 3 among children and adolescents, with sensitivity and specificity similar to the criteria recently suggested to diagnose MetS [31–33]. The current literature contains a plethora of trials and studies that established the clinical usefulness of TG/HDL-C ratio in distinguishing between children and adolescents with MetS [34], predicting coronary artery disease (CAD), peripheal artery disase (PAD) and crebrovascular accident (CVA) in pediatric and adolescent MetS [35] Few studies in Black Africa have focused CVD on adults and fewer still in adolescents. Yet, the link between TG/HDL-c ratio and cardiovascular risk factors among adolescent Black African, especially Nigerians, remains unknown. Therefore, the objective of this study was to investigate the relationship between TG/HDL-c ratio and cardiovascular risk factors among this group of people. The study also attempted to evaluate the optimal cutoff points of TG/HDL-c ratio to predict cardiovascular risk factors in Nigerian early, mid- and late adolescents.

Materials and methods

Sample design

Detailed description of the study population and the methods used in conducting the study have already been extensively described in a previous publication [18]. However, a brief clarification is rendered below. The study, descriptive and cross-sectional, was conducted at Biochemistry and Nutrition Department of the Nigerian Institute of Medical Research (NIMR), Lagos, Nigeria. In the study, eligible adolescents aged 10–19 years, were selected from different secondary schools for a dietary and nutrition survey (DNS) and primary data were collected from them. Originally, 624 participants (241 boys and 383 girls) were recruited but the data of only 613 (98.2%) were processed and analyzed in this article because of incomplete questionnaire responses. On the 4th of February 2019, the Institutional Review Board of the

Nigerian Institute of Medical Research (NIMR IRB (IRB/18/062) approved the study protocol, and the study was carried out in accordance with the Declaration of Helsinki (2000). Recruitment of participants into the study started on Thursday, October 10, 2019 (10/10/19) and ended on Friday, March 20, 2020 (03/20/20). The purpose of the study was clearly explained to each participating student. Each student was given an ample opportunity to ask questions for better understanding of the study. Thereafter, students who, in the presence of their teachers, gave verbal assent to participate in the study, were then given consent forms for their parents to also read, understand and sign or thumbprint.

Study site. This study was conducted in metropolitan Lagos, Lagos State, southwest Nigeria, a city-state, an economically viable and most populous city in the country. When this study was conducted, there were 616 registered public (372, 60.4%) and private (244, 39.6%) secondary schools in the local government areas (LGAs) selected for the study [36].

Study population. These were students aged 10–19 year who were attending government-authorized secondary schools in Lagos State Nigeria. The age range between 10–19 years had been defined by the World Health Organization (WHO) as the period of adolescence [37].

Sample size. The sample size, sampling technique and procedure for the study have also been reported in an earlier publication [38]. Simply stated, the sample size was determined for a specific population with 95% confidence interval, 54% proportion, a margin of error 5%, and accepting 12% non-response. The sample size was set at 650 students to guarantee that results of the study are expressive of majority of Nigerian ethnic groups inhabitant in Lagos State, and to cater for attrition and lost data. Flowchart of participants' recruitment into the study is illustrated in Fig 1.

Eligibility criteria. To be eligible, participants must be 10–19 years of age, dark-skinned and indigenous citizen of Nigeria. He or she must be resident in the community for a minimum of two years in the respective area of the study, must also be a regular student at any of the selected secondary schools. The State Ministry of Education and parents must

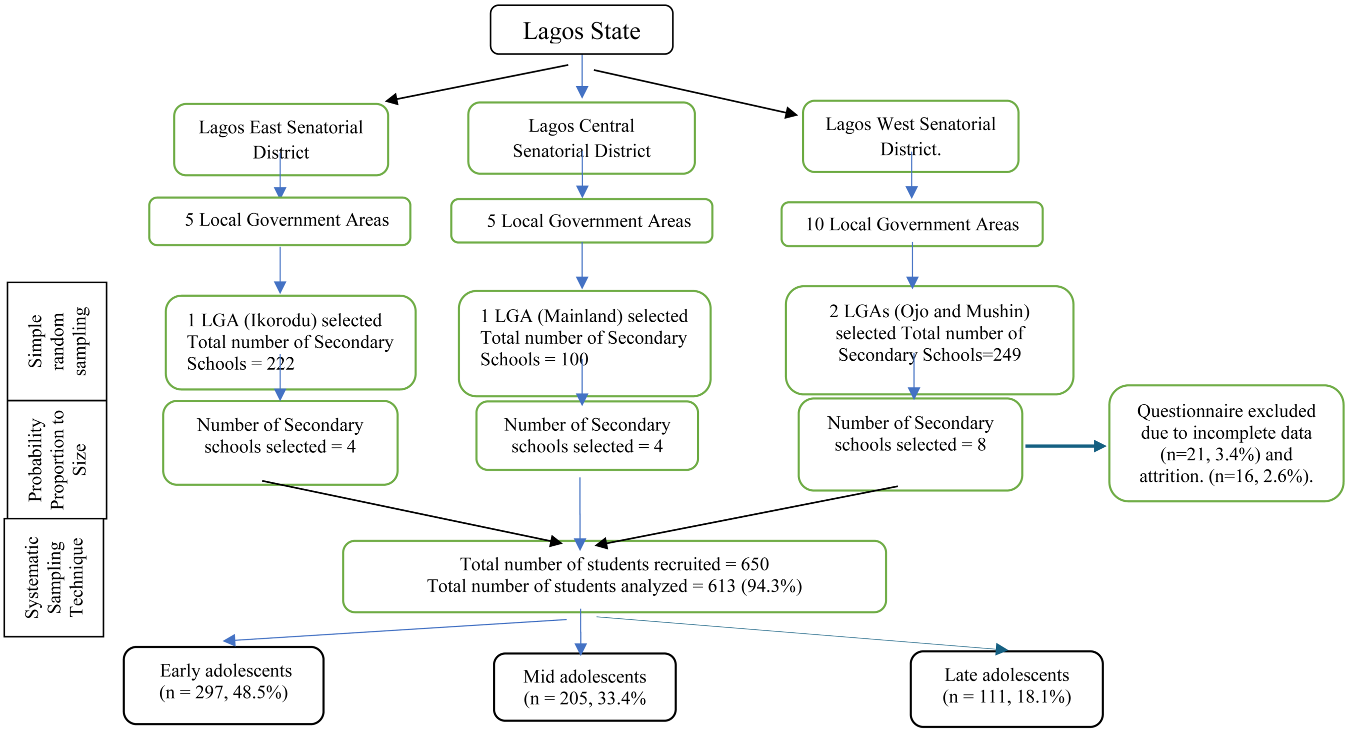

Fig 1. Flowchart of recruitment of study participants.

https://doi.org/10.1371/journal.pone.0322266.g001

give their documented approvals for the study and each student must also be willing to participate. Students who were (i) on therapeutic diet or drugs (ii) on admissions to a health facility in previous 6 months, (iii) known diabetics (iv) having vascular/liver/renal or any other chronic illness and those taking lipid-lowering medications were excluded. Also excluded were females who were (i) pregnant (ii) suspected to be pregnant (iii) breastfeeding and (vi) those on oral contraceptives.

Questionnaire: A semi-structured questionnaire was administered to record socio-demographic, dietary and nutritional data and to record anthropometric measurements and readings of systolic and diastolic blood pressure. These and all other laboratory procedures have earlier been documented [38].

Statistical analysis: In this report, participants were segregated by stage of adolescence (early or 10–13 years of age, mid or 14–17 years of age and late or 18–19 years of age). The data was subjected to descriptive statistics, Analysis of variance and ROC analysis using NCSS version 22 (Kaysville, Utah, USA). The sensitivity and specificity of each TG/HDL level for the detection of MetS, systolic and diastolic hypertension, diabetes, hypertriglyceridemia, hypercholesterolemia, low high-density lipoprotein cholesterol, high low-density lipoprotein cholesterol and ≥2 of these risk factors were calculated by creating dichotomous cardiometabolic variables for each TG/HDL-c value. Furthermore, the cutoff point, distance on the receiver operating characteristic (ROC) curve, sensitivity and specificity of each TG/HDL value was calculated using the NCSS statistical software. The TG/HDL-c value with the shortest distance on the ROC curve was considered in the determination of optimal cutoff for the identification of any of the cardiometabolic risk factors. The overall performance of the TG/HDL-c test for distinguishing cardiovascular risk factors was rated by calculating the area under the curves (AUC) in which an AUC of 1 was regarded to hold a flawless discriminatory power, and an AUC of 0.5 or below to submit that the discriminatory power is just accidental. Values were reported as mean (±), standard deviation (SD) and 95% CI for the continuous variables. Shapiro-Wilk's Normality test for normality of data distribution for continuous measures was conducted and when the test failed (i.e., p-value was <0.05, the normality of data distribution was rejected, and Kruskal-Wallis ANOVA was used to determine differences between 2 or more medians. An unadjusted p-value <0.05 was considered statistically significant. Data were illustrated as tables, graphs, charts, and figures.

Definitions. This has also been earlier documented [38]. Briefly, unacceptable lipid levels were classified as (i) total cholesterol ≥200 mg/dL (ii) low-density lipoprotein-cholesterol (LDL-c) ≥ 130 mg/dL (iii) triglycerides (TG) ≥130 mg/dL (hypertriglyceridemia), (iv) high-density lipoprotein cholesterol (HDL-c) < 40 mg/dL [39]. (v) Fasting plasma glucose (FPG) of ≥126 mg/dL was taken as diabetic; (vi) stage 1 hypertension, BP 130–139/80–89, and stage 2 ≥ 140/90 mmHg [40]. Triglyceride/High-density lipoprotein-c (TG/HDL-c) is the ratio of triglyceride divided by high-density lipoprotein-c of each study participant.

Results

Socio demographic and anthropometric characteristics of the study subjects, measurements, laboratory analyses as well as the prevalence of metabolic syndrome, have already been documented extensively in previous publications [41]. As presented in Table 1, the overall median TG/HDL-c ratio was significantly higher (P-value=<0.00001) in adolescents with (n = 52, 8.5%; median = 8.2), compared to those without (n = 561, 91.5%: median = 3.0), MetS. This significance is also evident in each stage of adolescence – early, mid and late, of the study subjects. On the other hand, overall median TG/HDL-c ratio among all subjects with either systolic or diastolic hypertension and those with diabetic fasting plasma glucose were evenly distributed, regardless of their stage of adolescence. Table 2 highlights age-standardized cardiovascular disease risk factors – SBP, DBP, FPG, TG, TC, HDL-c and LDL-c, relative to TG/HDL-c category in all the study subjects and in different stages of adolescence. With an increasing TG/HDL-c, plasma total cholesterol increased from 27.2 mg/dL among those with TG/HDL-c ≤ 1.0 to 223.4 mg/dL in those with TG/HDL-c > 5.0, plasma HDL-c decreased from 123.6 mg/dL to 48.6 mg/dL, though these changes did not reach any level of significance. In early adolescence, plasma TC increased with an increase in TG/HDL-c ratio from 36.1 among subjects with TG/HDL-c ratio of ≤1.0 to 213.8 mg/dL in those with TG/HDL-c > 5.0 while plasma HDL-c decreased from 117.0 to 49.1 mg/dL. Table 3 illustrates age-standardized

Table 1. Median values of TG/HDL-c ratio relative to cardiometabolic risk factors in Nigeria adolescents.

Vari-ables	Metabolic syndrome		Cardiometabolic risk factors													
			Hypertension				Diabetic FPG		Hypertriglyceri-demia		Hypercholester-olemia		Low HDL-c		High LDL=c	
			Systolic		Diastolic											
All (n=613)	Yes (n=52, 8.5%)	No (n=561, 91.5%)	Yes (n=109, 17.8%)	No (n=50482.2%)	Yes (n=8, 1.3%)	No (n=605, 98.7%)	Yes (n=71, 11.6%)	No (n=542, 88.4%)	Yes (n=431, 70.3%)	No (n=182, 29.7%)	Yes (n=262, 42.7%)	No (n=351, 57.3%)	Yes (n=153, 25.0%)	No (n=460, 75.0%)	Yes (n=525, 85.6%)	No (n=88, 14.4%)
	8.2	3.0	3.3	3.2	3.1	3.2	3.1	3.3	4.2	1.3	5.3	2.8	7.3	2.5	3.3	1.8
	52.2 (<0.00001)*		0.0001 (>0.05)		0.05 (>0.05)		1.63 (>0.05)		200.7 (<0.00001)		119.8 (<0.00001)		58.8 (<0.00001)		14.7 (0.0001)	
Early adoles-cents	Yes (n=24, 46.2%)	No (n=273, 48.7%)	Yes (n=52, 49.5%)	No (n=245, 48.6%)	Yes (n=4, 50.0%)	No (n=293, 48.4%)	Yes (n=44, %)	No (n=253, %)	Yes (n=201, 46.6%)	No (n=96, 52.8%)	Yes (n=112, 42.7%)	No (n=185, 52.7%)	Yes (n=70, 45.7%)	No (n=227, 49.4%)	Yes (n=257, 49.0%)	No (n=40, 45.5%)
	6.4	2.9	2.6	3.1	1.3	3.0	3.0	3.0	3.9	1.2	4.4	2.1	6.7	2.4	3.1	1.8
	16.3 (<0.00001)*		0.77 (>0.05)		0.90 (>0.05)		0.27 (>0.05)		108.1 (<0.00001)		51.2 (<0.00001)		24.8 (<0.00001)		12.5 (0.0004)	
Mid-adoles-cents	Yes (n=21, 40.4%)	No (n=184, 32.8%)	Yes (n=40, 36.7%)	No (n=16532.7%)	Yes (n=2, 25.0%)	No (n=203, 33.6%)	Yes (n=16, %)	No (n=189, %)	Yes (n=150, 34.8%)	No (n=55, 30.2%)	Yes (n=99, 37.8%)	No (n=106, 30.2%	Yes (n=54, 35.3%)	No (n=151, 32.8%)	Yes (n=174, 33.1%)	No (n=31, 35.2%)
	9.4	3.3	3.9	3.7	3.3	3.8	3.5	3.7	4.9	1.3	6.2	1.9	7.7	2.7	3.8	2.7
	28.6 (<0.00001)*		0.41 (>0.05)		0.06 (>0.05)		0.47 (>0.05)		75.5 (<0.00001)		47.8 (<0.00001)		23.0 (<0.00001)		2.21 (0.14)	
Late adoles-cents	Yes (n=7, 13.5%)	No (n=104, 18.5%)	Yes (n=17, 15.6%)	No (n=94, 18.7%)	Yes (n=2, 25.0%)	No (n=109, 18.0%)	Yes (n=11, %)	No (n=100, %)	Yes (n=80, 18.6%)	No (n=31, 17.0%)	Yes (n=51, 19.5%)	No (n=60, 17.1%)	Yes (n=29, 19.0%)	No (n=82, 17.8%)	Yes (n=94, 17.9%)	No (n=17, 19.3%)
	11.3	3.2	3.3	3.4	6.1	3.3	3.1	3.4	4.2	1.2	5.5	1.9	9.8	2.6	3.4	1.8
	8.2 (<0.00001)*		0.01 (>0.05)		0.67 (>0.05)		0.41 (>0.05)		19.4 (0.00001)		19.7 (0.00001)		10.3 (0.001)		1.87 (0.17)	

*Kruskal-Wallis ANOVA (P-value); H0=All medians are equal; H1=At least two medians are different. Yellow highlight=Reject H0

https://doi.org/10.1371/journal.pone.0322266.t001

Table 2. Age-standardized cardiovascular disease risk factors relative to TG/HDL-c category in different stages of adolescence in Nigeria.

Stages of adolescence	Cardiovascular risk factors	TG/HDL-c ratio category						P-value
		≤1.0	>1.0 to ≤2.0	>2.0 to ≤3.0	>3.0 to ≤4.0	>5.0 to ≤5.0	>5.0	
All (n = 613)	Population (%)	16 (2.6)	51 (8.3)	75 (12.2)	51 (8.3)	48 (7.8)	372 (60.7)	–
	Age (yrs)	14.2 (2.5)	14.7 (2.1)	14.7 (2.0)	14.5 (2.2)	14.4 (1.9)	14.8 (2.2)	0.10
	Systolic blood pressure (SBP) mm Hg	101.5 (11.4)	107.9 (12.0)	109.2 (11.1)	103.4 (13.3)	106.7 (13.6)	109.2 (12.3)	0.33
	Diastolic blood pressure (DBP) mm Hg	58.6 (8.4)	64.7 (9.4)	66.2 (9.2)	64.4 (8.5)	65.6 (11.2)	67.0 (9.6)	0.0008
	Fasting plasma glucose (FPG) mg/dL	67.4	82.4	88.1	88.1	81.4	89.3	0.37*
	Triglyceride (TG) mg/dL	155.0	194.5	217.7	182.5	193.4	204.2	0.14*
	Total cholesterol (TC) mg/dL	27.2	77.6	105.5	139.7	154.0	223.4	0.14*
	High-density lipoprotein cholesterol (HDL-c) mg/dL	123.6	106.0	80.4	79.0	70.4	48.6	0.06*
	Low-density lipoprotein cholesterol (LDL-c) mg/dL	370.8	209.7	275.3	291.1	310.3	291.1	0.26*
Metabolic syndrome (Freq. (%))		0 (0.0)	1 (2.0)	0 (0.0)	2 (3.9)	0 (0.0)	49 (13.2)	–
Early adolescents (n = 297)	Population (%)	9 (3.0)	25 (8.4)	35 (11.8)	29 (9.8)	28 (9.4)	171 (57.6)	–
	Age (yrs)	12.4 (1.5)	13.0 (1.2)	13.0 (1.1)	13.0 (1.5)	13.2 (1.3)	12.9 (1.3)	0.13
	Systolic blood pressure (SBP) mm Hg	104.6 (12.2)	106.7 (11.3)	105.4 (11.0)	103.4 (12.4)	104.1 (11.4)	108.1 (13.0)	0.79
	Diastolic blood pressure (DBP) mm Hg	59.2 (7.2)	65.4 (8.5)	64.3 (8.8)	65.3 (8.6)	62.6 (9.2)	66.7 (9.8)	0.0008
	Fasting plasma glucose (FPG) mg/dL	72.1	91.2	72.9	85.7	79.6	90.9	0.46*
	Triglyceride (TG) mg/dL	156.0	189.4	223.9	209.0	212.3	208.0	0.49*
	Total cholesterol (TC) mg/dL	36.1	77.6	104.3	133.1	166.5	213.8	0.34*
	High-density lipoprotein cholesterol (HDL-c) mg/dL	117.0	106.0	84.5	74.5	71.3	49.1	0.22*
	Low-density lipoprotein cholesterol (LDL-c) mg/dL	359.9	135.2	291.1	300.3	331.9	295.1	0.39*
Metabolic syndrome (Freq. (%))		0 (0.0)	1 (1.6)	0 (0.0)	2 (3.7)	0 (0.0)	21 (30.4)	–
Mid-adolescents (n = 205)	Population (%)	4 (25.0)	16 (31.4)	27 (36.0)	14 (27.5)	14 (29.2)	130 (34.9)	–
	Age (yrs)	15.9 (0.6)	15.7 (0.5)	15.6 (0.5)	15.7 (0.5)	15.2 (0.4)	15.6 (0.6)	0.20
	Systolic blood pressure (SBP) mm Hg	96.7 (9.7)	110.7 (15.1)	112.6 (8.7)	105.1 (14.8)	108.0 (16.2)	109.8 (12.4)	0.45
	Diastolic blood pressure (DBP) mm Hg	57.0 (10.1)	65.4 (12.2)	69.2 (8.7)	62.7 (9.5)	68.0 (11.3)	67.3 (10.1)	0.25
	Fasting plasma glucose (FPG) mg/dL	27.2	79.1	93.4	85.2	79.1	86.9	0.53*
	Triglyceride (TG) mg/dL	133.0	199.1	212.8	177.8	186.8	199.0	0.47*
	Total cholesterol (TC) mg/dL	65.6	81.0	108.9.4	136.0	128.2	231.6	0.31*
	High-density lipoprotein cholesterol (HDL-c) mg/dL	238.2	109.0	79.8	80.5	62.4	47.1	0.30*
	Low-density lipoprotein cholesterol (LDL-c) mg/dL	343.1	244.5	275.3	305.8	290.7	291.4	0.39*
Metabolic syndrome (Freq. (%))		0 (0.0)	0 (0.0)	0 (0.0)	1 (4.8)	3 (15.0)	17 (22.4)	–
Late adolescents (n = 111)	Population (%)	3 (18.7)	10 (19.6)	13 (17.3)	8 (15.7)	6 (12.5)	71 (19.1)	–
	Age (yrs)	17.4 (0.5)	17.7 (0.6)	17.7 (0.8)	17.9 (0.7)	17.8 (0.8)	17.8 (0.7)	0.40
	Systolic blood pressure (SBP) mm Hg	98.7 (11.5)	106.6 (7.2)	112.0 (13.4)	100.2 (15.3)	116.0 (14.7)	110.9 (10.4)	0.63
	Diastolic blood pressure (DBP) mm Hg	58.7 (12.9)	61.9 (6.5)	64.9 (10.0)	64.4 (7.0)	74.0 (15.3)	67.4 (7.9)	0.92
	Fasting plasma glucose (FPG) mg/dL	63.2	73.5	88.1	90.0	100.6	87.2	0.46*
	Triglyceride (TG) mg/dL	169.4	164.6	164.2	178.3	152.9	203.2	0.41*
	Total cholesterol (TC) mg/dL	13.9	65.4	103.0	176.8	170.9	233.9	0.38*
	High-density lipoprotein cholesterol (HDL-c) mg/dL	67.6	68.3	80.4	101.7	76.9	48.0	0.35*
	Low-density lipoprotein cholesterol (LDL-c) mg/dL	393.5	263.9	207.4	212.0	303.2	265.5	0.45*
Metabolic syndrome (Freq. (%))		0 (0.0)	0 (0.0)	0 (0.0)	1 (5.0)	1 (9.1)	5 (14.7)	–

*Kruskal-Wallis ANOVA.

https://doi.org/10.1371/journal.pone.0322266.t002

Table 3. Age-specific prevalence of risk factors by TG/HDL-c category among Nigerian adolescents.

Stages of adolescence	Cardiovascular risk factors	TG/HDL-c ratio category						P-svalue
		≤1.0	>1.0 to ≤2.0	>2.0 to ≤3.0	>3.0 to ≤4.0	>5.0 to ≤5.0	>5.0	
All (n=613)	Population (%)	16 (2.6)	51 (8.3)	75 (12.2)	51 (8.3)	48 (7.8)	372 (60.7)	–
	Systolic hypertension	3 (18.7)	8 (15.7)	13 (17.3)	9 (17.6)	9 (18.7)	67 (18.0)	1.00
	Diastolic hypertension	0 (0.0)	0 (0.0)	3 (4.0)	0 (0.0)	0 (0.0)	5 (1.3)	0.27
	Diabetic FPG	1 (6.2)	12 (23.5)	6 (8.0)	7 (13.7)	6 (12.5)	39 (10.5)	0.10
	Hypertriglyceridemia	0 (0.0)	8 (15.7)	19 (25.3)	31 (60.8)	30 (62.5)	343 (92.2)	<0.00001
	Hypercholesterolemia	0 (0.0)	0 (0.0)	5 (6.7)	11 (21.6)	16 (33.3)	230 (61.8)	<0.00001
	Low HDL	0 (0.0)	3 (5.9)	2 (2.7)	4 (7.8)	5 (10.4)	139 (37.4)	<0.00001
	High LDL	11 (68.7)	33 (64.7)	62 (82.7)	40 (78.4)	46 (95.8)	333 (89.5)	<0.00001
Early adolescents (n = 297)	Population (%)	9 (3.0)	25 (8.4)	35 (11.8)	29 (9.8)	28 (9.4)	171 (57.6)	–
	Systolic hypertension	2 (22.2)	4 (16.0)	7 (20.0)	6 (20.7)	6 (21.4)	27 (15.8)	0.95
	Diastolic hypertension	0 (0.0)	0 (0.0)	3 (8.6)	0 (0.0)	0 (0.0)	1 (0.6)	0.008
	Diabetic FPG	0 (0.0)	7 (28.0)	4 (11.4)	4 (13.8)	5 (17.9)	24 (14.0)	0.34
	Hypertriglyceridemia	0 (0.0)	2 (8.0)	7 (20.0)	17 (58.6)	18 (64.3)	157 (91.8)	<0.00001
	Hypercholesterolemia	0 (0.0)	0 (0.0)	1 (2.9)	6 (20.7)	10 (35.7)	95 (55.6)	<0.00001
	Low HDL	0 (0.0)	3 (12.0)	1 (2.9)	2 (6.9)	3 (10.7)	61 (35.7)	<0.00001
	High LDL	6 (66.7)	13 (52.0)	31 (88.6)	23 (79.3)	28 (100.0)	156 (91.2)	<0.00001
Mid-adolescents (n = 205)	Population (%)	4 (25.0)	16 (31.4)	27 (36.0)	14 (27.5)	14 (29.2)	130 (34.9)	–
	Systolic hypertension	1 (25.0)	4 (25.0)	5 (18.5)	0 (0.0)	1 (7.1)	29 (22.3)	0.33
	Diastolic hypertension	0 (0.0)	0 (0.0)	0 (0.0)	0 (0.0)	0 (0.0)	2 (1.5)	0.95
	Diabetic FPG	0 (0.0)	3 (18.7)	2 (7.4)	2 (14.3)	0 (0.0)	9 (6.9)	0.40
	Hypertriglyceridemia	0 (0.0)	3 (18.7)	8 (29.6)	8 (57.1)	6 (42.9)	125 (96.2)	<0.00001
	Hypercholesterolemia	0 (0.0)	0 (0.0)	3 (11.1)	2 (14.3)	4 (28.6)	90 (69.2)	<0.00001
	Low HDL	0 (0.0)	0 (0.0)	0 (0.0)	1 (7.1)	2 (14.3)	51 (39.2)	<0.00001
	High LDL	2 (50.0)	13 (81.2)	21 (77.8)	12 (85.7)	12 (85.7)	114 (87.7)	0.32
Late adolescents (n = 111)	Population (%)	3 (18.7)	10 (19.6)	13 (17.3)	8 (15.7)	6 (12.5)	71 (19.1)	–
	Systolic hypertension	0 (0.0)	0 (0.0)	1 (7.7)	3 (37.5)	2 (33.3)	11 (15.5)	0.19
	Diastolic hypertension	0 (0.0)	0 (0.0)	0 (0.0)	0 (0.0)	0 (0.0)	2 (5.9)	0.95
	Diabetic FPG	1 (33.3)	2 (10.0)	0 (0.0)	1 (12.5)	1 (16.7)	6 (8.5)	0.42
	Hypertriglyceridemia	0 (0.0)	3 (30.0)	4 (30.8)	6 (75.0)	6 (100.0)	61 (85.9)	<0.00001
	Hypercholesterolemia	0 (0.0)	0 (0.0)	1 (7.7)	3 (37.5)	2 (33.3)	45 (63.4)	<0.00001
	Low HDL	0 (0.0)	0 (0.0)	1 (7.7)	1 (12.5)	0 (0.0)	27 (38.0)	0.01
	High LDL	3 (100.0)	7 (70.0)	10 (76.9)	5 (62.5)	6 (100.0)	63 (88.7)	0.17

SHT= Systolic hypertension; DHT=Diastolic hypertension; FPG=Fasting Plasma Glucose.

https://doi.org/10.1371/journal.pone.0322266.t003

prevalence of risk factors by TG/HDL-c category. Table 3 illustrates the age-specific prevalence of risk factors by TG/HDL-c ratio among the study subjects. Overall, the prevalence of hypertriglyceridemia and hypercholesterolemia significantly increased (P-value<0.00001) from 0.0 and 0.0 to 92.2% and 61.8% respectively as the TG/HDL-c ratio increased. This trend was also evident in each of the three stages of adolescence. The prevalence of high LDL in mid- and late adolescents, relative to increase in TG/HDL-c ratio were not important. Table 4 illustrates the ROC curves, Z-value and P-value of AUC, cut-off point, sensitivity, specificity, and distance on the ROC curve for the detection of non-lipid cardiometabolic variables such as MetS, SHT, DHT, dFPG, for each of the early, mid- and late adolescence. An inverse relationship was observed between the stages of adolescence and cutoff for MetS (7.92, 7.31 and 5.10 respectively), SHT

Table 4. Sensitivity (Sens), specificity (Spec), and distance in the receiver operating characteristic (ROC) curve for TG/HDL-c cutoffs for MetS, SHT, DHT and dFPG among Nigerian adolescents.

Area Under the Curve (AUC)												
**Statistics for ROC**	**Metabolic syndrome (Yes = 1; No = 0)**			**Systolic hypertension (Yes = 1; No = 0)**			**Diastolic hypertension (Yes = 1; No = 0)**			**Diabetic Fasting Plasma Glucose (Yes = 1; No = 0)**		
	**Adolescence**											
	**Early**	**Mid-**	**Late**	**Early**	**Mid-**	**Late**	**Early**	**Mid**	**Late**	**Early**	**Mid**	**Late**
AUC (Std Error)	0.87 (0.04)	0.82 (0.07)	0.75 (0.06)	0.53 (0.05)	0.51 (0.07)	0.46 (0.04)	0.45 (0.05)	0.67 (0.12)	0.36 (0.19)	0.45 (0.08)	0.44 (0.10)	0.48 (0.05)
Z-value (P-value)	9.92 (<0.00001)	4.44 (<0.00001)	4.36 (<0.00001)	0.61 (0.27)	0.11 (0.46)	−0.88 (0.81)	−1.07 (0.86)	0.91 (0.18)	−0.75 (0.77)	−0.65 (0.74)	−0.57 (0.72)	−0.51 (0.70)
Cutoff	≥7.92	≥7.31	≥5.10	≥2.94	≥1.76	≥1.14	≥2.85	≥3.25	≥8.37	≥0.72	≥5.0	≥0.55
Sensitivity (95% CL)	0.67 (0.43, 0.85)	0.71 (0.29, 0.96)	0.62 (0.41, 0.81)	0.70 (0.53, 0.83)	0.94 (0.71, 1.00)	0.88 (0.77, 0.97)	1.00 (0.16, 1.00)	1.00 (0.16, 1.00)	0.25 (0.01, 0.81)	1.00 (0.79, 1.00)	0.36 (0.11, 0.69)	1.00 (0.92, 1.00)
Specificity (95% CL)	0.90 (0.84, 0.94)	0.85 (0.76, 0.91)	0.81 (0.75, 0.85)	0.44 (0.36, 0.52)	0.30 (0.21, 0.40)	0.15 (0.11, 0.20)	0.41 (0.34, 0.48)	0.49 (0.39, 0.58)	0.91 (0.88, 0.94)	0.04 (0.02, 0.08)	0.70 (0.60, 0.79)	0.04 (0.02, 0.08)
Distance to corner	0.35	0.32	0.42	0.64	0.70	0.86	0.59	0.51	0.75	0.96	0.70	0.96

Agex1 = Early adolescents (Blue line); Agex2 = Mid-adolescents (Green line); Agex3 = Late adolescents (Red line); AUC = Area Under Curve; ROC = Receiver Operating Character; *H0 (The hypothesis that Hypertension and TG/HDL-c are independent is not rejected).

https://doi.org/10.1371/journal.pone.0322266.t004

(2.94, 1.76 and 1.14 respectively) and DHT (2.85, 3.25 and 8.37 respectively). However, the ability of TG/HDL-c to predict MetS was stronger in mid-adolescents with AUC (std. err) of 0.82 (0.07), Z value (P-value) of 4.44 (<0.00001), sensitivity of 0.71 and specificity of 0.85, with the shortest distance to corner of 0.32. The TG/HDL-c ratio was unreliable in predicting systolic hypertension (cutoff ≥2.94, distance to corner = 0.64), diastolic hypertension (cutoff ≥2.85, distance to corner = 0.59), or diabetic fasting plasma glucose (cutoff ≥0.72, distance to corner = 0.96) in early adolescence or in any other stage of adolescence. Table 5 illustrates the sensitivity, specificity, and distance in the receiver operating characteristic (ROC) curve for TG/HDL-c cutoffs for dyslipidemia – hypertriglyceridemia, hypercholesterolemia, low high-density lipoprotein cholesterol and high low-density-lipoprotein cholesterol among the study subjects. TG/HDL-c ratio had a very strong ability to predict hypertriglyceridemia, especially among those in early adolescents, with an AUC (Std Err) of 0.91 (0.02), sensitivity of 0.86, specificity of 0.91, cutoff of ≥2.40 and distance to corner of just 0.17. The TG/HDL-c ratio was weaker in forecasting hypertriglyceridemia in late adolescents (cutoff=≥2.37, sensitivity = 0.82, specificity = 0.83, and distance to corner = 0.24), and weakest in predicting hypertriglyceridemia in mid-adolescents (distance to corner = 0.39). On the other hand, TG/HDL-c ratio was strong in predicting hypercholesterolemia both in early adolescence with an AUC (Std Err) of 0.85 (0.03), sensitivity of 0.84, specificity of 0.78, cutoff of ≥3.64 and distance to corner of 0.27 as well as in mid-adolescence, with an AUC (Std Err) of 0.87 (0.03), sensitivity of 0.76, specificity of 0.88, cutoff of ≥3.98 and distance to corner of 0.26. TG/HDL-c was practically inconsequential in predicting low HDL-c in any of the three stages of adolescence but slightly stronger in forecasting high LDL-c especially in mid adolescents with an AUC (Std Err) of 0.45 (0.09), sensitivity of 0.76, specificity of 0.53, cutoff of ≥1.83 and distance to corner of 0.53. Thus, the discriminatory power of TG/HDL-c ratio for cardiovascular risk factors among adolescent Nigerians was mainly limited to predicting MetS, hypertriglyceridemia, and hypercholesterolemia especially in early and in mid-adolescents. Table 6 shows the sensitivity, specificity, and distance in the receiver operating characteristic (ROC) curve for TG/HDL-c cutoffs for total dyslipidemia and risk factors of two or more among Nigerian adolescents. In all the study subjects, the shortest distance on the ROC curve for

Table 5. Sensitivity (Sens), specificity (Spec), and distance in the receiver operating characteristic (ROC) curve for TG/HDL-c cutoffs for hypertriglyceridemia, hypercholesterolemia, low high-density lipoprotein cholesterol and high low-density-lipoprotein cholesterol among Nigerian adolescents.

Statistics for ROC	Hypertriglyceridemia (Yes = 1; No = 0)			Hypercholesterolemia (Yes = 1; No = 0)			Low High-density lipoprotein cholesterol (Yes = 1; No = 0)			High Low-density lipoprotein cholesterol (Yes = 1; No = 0)		
	Stages of adolescence											
	Early	Mid-	Late	Early	Mid-	Late	Early	Mid-	Late	Early	Mid-	Late
AUC (Std Error)	0.91 (0.02)	0.83 (0.04)	0.90 (0.02)	0.85 (0.03)	0.87 (0.03)	0.83 (0.02)	0.10 (0.02)	0.13 (0.04)	0.17 (0.03)	0.50 (0.07)	0.54 (0.09)	0.67 (0.05)
Z-value (P-value)	15.94 (<0.00001)	7.47 (<0.00001)	19.84 (<0.00001)	13.01 (<0.00001)	11.00 (<0.00001)	14.28 (<0.00001)	−16.47 (1.00)	−8.73 (1.00)	−10.40 (1.00)	0.23 (0.49)	0.45 (0.32)	3.22 (0.0006)
Cut-off	≥2.40	≥1.43	≥2.37	≥3.64	≥3.98	≥3.41	≥0.09	≥0.14	≥0.19	≥1.60	≥1.83	≥1.11
Sensitivity (95% CL)	0.86 (0.79, 0.91)	0.95 (0.88, 0.99)	0.82 (0.76, 0.87)	0.84 (0.75, 0.90)	0.76 (0.63, 0.87)	0.73 (0.64, 0.81)	1.00 (0.98, 1.00)	1.00 (0.96, 1.00)	1.00 (0.98, 1.00)	0.79 (0.73, 0.85)	0.76 (0.66, 0.84)	0.91 (0.87, 0.95)
Specificity (95% CL)	0.91 (0.80, 0.97)	0.61 (0.42, 0.78)	0.83 (0.74, 0.70)	0.78 (0.69, 0.86)	0.88 (0.77, 0.95)	0.79 (0.72, 0.85)	0.00 (0.00, 0.07)	0.00 (0.00, 0.12)	0.00 (0.00, 0.05)	0.42 (0.25, 0.61)	0.53 (0.28, 0.77)	0.45 (0.29, 0.62)
Distance to corner	0.17	0.39	0.24	0.27	0.26	0.34	1.00	1.00	1.00	0.62	0.53	0.56

Agex1 = Early adolescents (Blue line); Agex2 = Mid-adolescents (Green line); Agex3 = Late adolescents (Red line); AUC = Area Under Curve; ROC = Receiver Operating Character; *H0 (The hypothesis that Hypertension and TG/HDL-c are independent is not rejected).

https://doi.org/10.1371/journal.pone.0322266.t005

Table 6. Sensitivity (Sens), specificity (Spec), and distance in the receiver operating characteristic (ROC) curve for TG/HDL-c cutoffs for dyslipidemia and risk factors of two or more among Nigerian adolescents.

Statistics for ROC	Dyslipidemia (Yes = 1; No = 0)				Risk factors ≥2 (Yes = 1; No = 0)			
	Stages of adolescence							
	All	Early	Mid-	Late	All	Early	Mid-	Late
AUC (Std Error)	0.87 (0.02)	0.82 (0.03)	0.88 (0.03)	0.90 (0.02)	0.81 (0.02)	0.84 (0.03)	0.77 (0.04)	0.80 (0.03)
Z-value (P-value)	23.58 (<0.00001)	10.02 (<0.0001)	11.85 (<0.00001)	20.32 (<0.00001)	17.44 (<0.00001)	12.52 (<0.00001)	5.92 (. < 0.00001)	11.83 (<0.00001)
Cut-off	≥5.23	≥5.65	≥7.09	≥5.23	≥3.30	≥5.57	≥7.29	≥3.31
Sensitivity (95% CL)	0.94 (0.84, 0.99)	0.95 (0.75, 1.00)	1.00 (0.59, 1.00)	0.92 (0.74, 0.99)	0.76 (0.70, 0.81)	0.67 (0.56, 0.77)	0.41 (0.29, 0.56)	0.72 (0.63, 0.79)
Specificity (95% CL)	0.78 (0.75, 0.82)	0.74 (0.64, 0.80)	0.82 (0.73, 0.87)	0.84 (0.79, 0.88)	0.71 (0.66, 0.76)	0.89 (0.83, 0.94)	0.98 (0.91, 1.00)	0.77 (0.70, 0.83)
Distance to corner	0.22	0.26	0.18	0.18	0.37	0.35	0.59	0.37

Agex1 = Early adolescents (Blue line); Agex2 = Mid-adolescents (Green line); Agex3 = Late adolescents (Red line); AUC = Area Under Curve; ROC = Receiver Operating Character; *H0 (The hypothesis that Hypertension and TG/HDL-c are independent is not rejected).

https://doi.org/10.1371/journal.pone.0322266.t006

predicting dyslipidemia was 0.22, which lies between 0.18 (the shortest distance for predicting dyslipidemia in mid-adolescents and late adolescents respectively) and 0.26 (the shortest distance in early adolescents). The cutoff in early, mid- and late adolescents were 5.65, 7.09 and 5.23 respectively. The discriminatory power of TG/HDL-c ratio to predict ≥2 cardiovascular risk factors in all the study subjects was weaker, with the shortest distance on the ROC curve as 0.37, similar in late adolescents but lies between 0.35 in early and 0.59 in late adolescents. Table 7 illustrates the population distribution of each TG/HDL-c ratio and the sensitivity, specificity, and distance on the ROC curve for the detection of

Table 7. Sensitivity (Sens), specificity (Spec), and distance in the receiver operating characteristic (ROC) curve for each TG/HDL-c cutoff in adolescent Nigerians.

TG/HDL-c ratio		Per-centile	Diabetic FPG			Hypertriglyceridemia			Hypercholesterolemia			Low HDL-c			High LDL-c			≥2 risk factors		
			Sen. (%)	Spec. (%)	Dist. ROC curve	Sen. (%)	Spec. (%)	Dist. ROC curve	Sen. (%)	Spec. (%)	Dist. ROC curve	Sen. (%)	Spec. (%)	Dist. ROC curve	Sen. (%)	Spec. (%)	Dist. ROC curve	Sen. (%)	Spec. (%)	Dist. ROC curve
All	≤1.0	2.6	100.0	0.0	0.93	–	–	–	–	–	–	–	–	–	100.0	0.0	1.00	–	–	–
	>1.0 to ≤2.0	8.3	16.7	89.7	0.83	87.5	0.30	0.71	–	–	–	83.3	66.7	0.37	3.0	100.0	0.97	20.0	91.3	0.80
	>2.0 to ≤3.0	12.2	66.7	42.0	0.66	52.6	91.1	0.48	40.0	98.6	0.60	31.5	100.0	0.68	54.8	61.5	0.59	28.6	89.7	0.72
	>3.0 to ≤4.0	8.3	100.0	34.1	0.66	35.5	80.0	0.68	90.9	27.5	0.73	59.6	75.0	0.48	47.5	72.7	0.59	91.7	23.1	0.77
	>4.0 to ≤5.0	7.8	100.0	28.6	0.71	80.0	55.6	0.49	87.5	53.1	0.49	51.2	80.0	0.53	69.6	100.0	0.30	38.5	80.0	0.65
	>5.0	60.7	100.0	9.6	0.90	75.5	65.5	0.42	80.9	62.0	0.43	100.0	0.0	1.00	94.3	15.4	0.85	58.0	79.7	0.47
Early adolescence	≤1.0	3.0	–	–	–	–	–	–	–	–	–	–	–	–	16.7	100.0	0.83	–	–	–
	>1.0 to ≤2.0	8.4	100.0	11.1	0.89	100.0	34.8	0.65	–	–	–	81.8	66.7	0.40	23.1	83.3	0.79	33.3	90.9	0.67
	>2.0 to ≤3.0	11.8	100.0	3.2	0.97	57.1	89.3	0.44	100.0	0.0	0.0	47.1	100.0	0.53	90.3	75.0	0.27	75.0	51.6	0.54
	>3.0 to ≤4.0	9.8	25.0	100.0	0.75	52.9	83.3	0.50	66.7	56.5	0.55	40.7	100.0	0.59	30.4	83.3	0.71	57.1	63.6	0.56
	>4.0 to ≤5.0	9.4	60.0	65.2	0.53	100.0	30.0	0.70	90.0	50.0	0.51	96.0	33.3	0.67	–	–	–	100.0	18.2	0.82
	>5.0	57.6	100.0	12.2	0.88	71.3	71.4	0.40	76.8	67.1	0.40	100.0	0.0	1.00	93.6	20.0	0.80	53.4	80.9	0.50
Mid adolescence	≤1.0	25.0	–	–	–	–	–	–	–	–	–	–	–	–	100.0	0.0	1.00	–	–	–
	>1.0 to ≤2.0	31.4	33.3	100.0	0.67	100.0	38.5	0.62	–	–	–	–	–	–	15.4	100.0	0.85	–	–	–
	>2.0 to ≤3.0	36.0	100.0	36.0	0.64	37.5	94.7	0.63	33.3	100.0	0.67	–	–	–	61.9	66.7	0.51	100.0	36.0	0.64
	>3.0 to ≤4.0	27.5	100.0	58.3	0.42	100.0	33.3	0.67	100.0	25.0	0.75	46.1	100.0	0.54	100.0	100.0	0.00	100.0	41.7	0.58
	>4.0 to ≤5.0	29.2	–	–	–	66.7	100.0	0.33	100.0	70.0	0.30	41.7	100.0	0.58	58.3	100.0	0.42	75.0	70.0	0.39
	>5.0	34.9	88.9	27.3	0.74	96.0	60.0	0.40	90.0	45.0	0.56	100.0	0.0	1.00	95.6	12.5	0.88	74.3	76.8	0.35
Late adolescence	≤1.0	18.7	100.0	0.0	1.0	–	–	–	–	–	–	–	–	–	–	–	–	–	–	–
	>1.0 to ≤2.0	19.6	50.0	75.0	0.56	100.0	14.3	0.86	–	–	–	–	–	–	71.4	33.3	0.73	100.0	12.5	0.87
	>2.0 to ≤3.0	17.3	–	–	–	100.0	77.8	0.22	100.0	58.3	0.42	25.0	100.0	0.75	40.0	100.0	0.60	100.0	8.3	0.92
	>3.0 to ≤4.0	15.7	100.0	57.1	0.43	100.0	50.0	0.50	33.3	100.0	0.67	85.7	100.0	0.14	40.0	100.0	0.60	66.7	60.0	0.52
	>4.0 to ≤5.0	12.5	100.0	60.0	0.40	–	–	–	50.0	100.0	0.50	–	–	–	–	–	–	66.7	55.7	0.47
	>5.0	19.1	66.7	53.9	0.57	55.7	90.0	0.45	86.7	73.1	0.30	100.0	0.0	1.00	96.8	12.5	0.88	50.0	96.5	0.50

https://doi.org/10.1371/journal.pone.0322266.t007

hypertension, dyslipidemia, diabetes, and ≥2 of these risk factors for all study subjects and for each of the three stages of adolescence, respectively.

Discussion

This study provides age-specific-based data on the optimal cutoff of the triglyceride to HDL cholesterol (TG/HDL-c) ratio for detecting cardiovascular risk factors among a sub-Sahara African population in different stages of adolescence, aged between 10 and 19 years. To the best of the authors' knowledge, this may be the first cross-sectional study to evaluate the TG/HDL-c ratio cutoff among this group of people in this specific part of the globe. The study also reports an association between the TG/HDL-c ratio and cardiovascular risk factors. In sub-Saharan Africa, TG/HDL-c is a rather novel atherogenic tool for predicting MetS and other cardiometabolic variables, and the reports on this in the Nigerian adolescent population are rare. To detect MetS, the National Cholesterol Education Program (NCEP) Adult Treatment Panel III (ATPIII) standards registered optimal cutoff for TG/HDL-c as 1.6 and 1.2 in men and 1.1 in women, respectively [42]. Studies in China described the cutoff values of TG/HDL-c to identify MetS as 0.88 [43] and 0.84 in Zhuhai women [44]. In an African study, the cutoff value in Ghanaian women was reported as 0.61 [45] and in Canadian men and women, it was reported as 1.62 and 1.18, respectively [46]. In contrast to these reports, in the present study, consequent upon the sensitivity, specificity, and ROC calculations, the optimal cutoffs of TG/HDL-c ratio for Nigerian adolescents were 7.92 in early adolescence, 7.31 in mid adolescence and 5.10 in late adolescence respectively. The reasons for these high cutoff points among Nigerian adolescents are not understood. Future studies may be able to elucidate probable nutritional, dietary, environmental, climatic, living standard, genetic or even hereditary basis for the variation in these cutoffs in different population groups. At this point, it can only be speculated that these cutoffs are relatively high in adolescents because the risk factor for MetS and cardiovascular diseases are in the formative stages. In adolescence, TG,HDL-c has a weak predictive value for systolic and diastolic hypertension and failed to forecast diabetic fasting blood glucose in Nigerian adolescents. This may suggest that systolic hypertension, (and less so diastolic hypertension) may not necessarily be an integral aspect of MetS. Although studies have linked the occurrence of MetS to diabetes and insulin resistance [47–49], a study reported that TG/HDL-c ratio is not a reliable tool as marker of insulin resistance, especially in African Americans [50], which resonates with the findings of this study. It should be noted however, that a contrary view is held by Baneu et al, who stated that the predictive power of TG/HDL ratio varies across ethnicities and sexes, with specific thresholds providing greater accuracy for Caucasians, Asians, and Hispanics over African Americans and for men over women [51]. Evidence also abound of variations in racial and ethnic background of the actual values of the ratio that best identifies individuals [48,50,52]. It is also noted that variations in the optimal values of TG/HDL-c ratio between populations might be due to varied body size, physical activity, metabolic status [43] and age difference. Additionally, this study showed an increasing trend in the prevalence of total cholesterol and a decreasing trend in high-density lipoprotein with higher TG/HDL-c in all the adolescents when they were not segregated into the three stages. Both increasing and decreasing trends were observed in total cholesterol and high-density lipoprotein with increasing TG/HDL-c in early adolescents but not mid- or late adolescence. The reason for this is unclear but probably indicates the initial stage of atherogenic risk factors as basis for future variables such as hypertension and insulin resistance. Dharuni et al suggested that the elements that comprise metabolic syndrome are separately related with later higher risk for all-cause mortality, particularly cardiovascular disease [53]. Nonetheless, this study evaluated TG/HDL-c ratio in the framework of cardiometabolic risk expectation. Thus, data from this study shows that the TG/HDL-c ratio is a meaningful predictor of cardiometabolic risk factors in this adolescent population. The inverse relationship between stages of adolescence and TG/HDL-c cutoff for predicting MetS suggests a gradual development of cardiometabolic component of MetS which may be rarer in early but more frequent in late adolescents. The observed correlations between higher TG/HDL-c ratios and increased markers of individual cardiometabolic risk, such as hypertriglyceridemia, hypercholesterolemia emphasize the significance of this ratio as a principal indicator of potential health risks in adolescence. Studies have shown that hypertriglyceridemia is associated with a prolonged risk of atherosclerotic cardiovascular disease (ASCVD) [54]. Unchecked mild-to moderate hypertriglyceridemia of 150–499 mg/dL concentration could lead to severe form of >880 mg/dL and, at a higher concentration

of >1770mg/dL, to acute pancreatitis, a clinically important risk [55] and multiorgan failure [56]. In this study, the median value of TG/HDL-c ratio among adolescents with hypertriglyceridemia (4.2) was significantly higher (P-value<0.00001) than that among those with normal triglyceride concentration (1.3). This is similar to the findings of Dharuni et al in in an adult population in South India [53] which reported that TG/HDL-c ratio is not only a strong independent predictor of myocardial infarction (MI) but also that of coronary heart disease (CHD). Da Luz et al, in a Brazilian study, reported that TG/HDL-c ratio exhibited the strongest link with coronary disease scored by Friesinger index [57] while Shou P et al, in a Chinese study on adults, showed that TG/HDL-c was the best prognostic factor of MetS in women whereas in men waist-hip ratio was the best predictor [58]. According to a Copenhagen Male Study, TG/HDL-c ratios can more precisely identify and distinguish coronary disease [59]. Edwards et al [60] and Brinton and his colleagues [61] suggested that the conceivable pathway is that high triglycerides and low HDL-c form the foundation for the buildup of small and dense LDL-c, causing HDL-c particles to initiate accelerated catabolism, thus concluding the atherogenic circle. The current study suggests that the cutoff of TG/HDL-c ratio is a good predictor of cardiometabolic risk factors, especially dyslipidemia but not systolic hypertension or diabetic fasting plasma glucose. With its high sensitivity and specificity, and it will be about the most appropriate means of predicting the incidence of current (in adolescence) and possible future (in adulthood) atherosclerotic diseases in Nigeria.

Strengths and limitations

This study specified facts and figures on cutoffs and AUC for specific atherogenic variables stratified by three stages of adolescence – early, mid and late. Other researchers, especially in sub-Saharan Black Africa can utilize the cutoffs proposed in this study for the screening and clinical intervention of cardiovascular disease especially in this age group. However, the study has some limitations that need further consideration. First, the sample size is relatively small. A larger sample size would probably have given a more robust analysis and extend its external validity. Second, bias may have been introduced in the sample selection as the proportion of girls outweighed that of boys, and some of the overweight or obese students may have been left out. Thirdly, the study was conducted in the southwest corner of Nigeria, on the Atlantic Ocean coastline thus it may not be representative of Nigerian adolescent population in the arid eco-system, the population living in high altitude region in north-central part of the country or those resident in the rain-forest zone. Fourthly, this was a cross-sectional study thus devoid of predicting any prognosis nor does it assume cause and effect of any findings. Finally, the study did not consider anthropometric status such as waist circumference, Body Mass index-for-age to determine lean, overweight or obese adolescents, sex, or ethnicity. It is expected that future studies will be able to recruit a much larger number of participants, probably in a national or multi-center study of TG/HDL-c.

Conclusions

To conclude, this study showed a TG/HDL-c ratio effectively predicted MetS in early, mid- and late adolescent stages with cutoff values of 7.92, 7.31 and 5.10; hypertriglyceridemia with cutoff of 2.40, 1.43 and 2.37 respectively; and hypercholesterolemia with cutoff of 3.64, 3.98 and 3.41 respectively to differentiate high cardiovascular risk adolescents. The study also documented the continuous relationship between two cardiovascular disease risk factors – total cholesterol and high-density lipoprotein in all the study subjects and TG/HDL-c ratio. As a tool, the TG/HDL-c ratio should be considered for the initial prediction of MetS and dyslipidemia.

Acknowledgments

Dr. Bamgboye M. Afolabi is the guarantor of this work and, as such, has full access to all the data in the study and takes responsibility for the integrity of the data and the accuracy of the data analysis. This project was supported by the Nigerian Institute of Medical Research (NIMR). We would like to thank the Lagos State Ministries of Education, Health and Public Service and their districts heads for making this study possible. We also thank the parents of the students as well as the students for making this study possible.

Author contributions

Conceptualization: Bamgboye M. Afolabi, Susan J Holdbrooke.

Data curation: Bamgboye M. Afolabi, Susan J Holdbrooke.

Formal analysis: Bamgboye M. Afolabi, Susan J Holdbrooke.

Investigation: Bamgboye M. Afolabi.

Methodology: Bamgboye M. Afolabi.

Project administration: Susan J Holdbrooke.

Resources: Susan J Holdbrooke.

Software: Bamgboye M. Afolabi.

Validation: Bamgboye M. Afolabi.

Writing – original draft: Bamgboye M. Afolabi.

Writing – review & editing: Bamgboye M. Afolabi.

References

1. Palmer AK, Jensen MD. Metabolic changes in aging humans: current evidence and therapeutic strategies. J Clin Invest. 2022;132(16):e158451. https://doi.org/10.1172/JCI158451 PMID: 35968789
2. Kennedy BK, Berger SL, Brunet A, Campisi J, Cuervo AM, Epel ES, et al. Geroscience: linking aging to chronic disease. Cell. 2014;159(4):709–13. https://doi.org/10.1016/j.cell.2014.10.039 PMID: 25417146
3. López-Otín C, Blasco MA, Partridge L, Serrano M, Kroemer G. The hallmarks of aging. Cell. 2013;153(6):1194–217. https://doi.org/10.1016/j.cell.2013.05.039 PMID: 23746838
4. Bechtold M, Palmer J, Valtos J, Iasiello C, Sowers J. Metabolic syndrome in the elderly. Curr Diab Rep. 2006;6(1):64–71. https://doi.org/10.1007/s11892-006-0054-3 PMID: 16522283
5. Denys K, Cankurtaran M, Janssens W, Petrovic M. Metabolic syndrome in the elderly: an overview of the evidence. Acta Clin Belg. 2009;64(1):23–34. https://doi.org/10.1179/acb.2009.006 PMID: 19317238
6. Moore JX, Chaudhary N, Akinyemiju T. Metabolic Syndrome Prevalence by Race/Ethnicity and Sex in the United States, National Health and Nutrition Examination Survey, 1988-2012. Prev Chronic Dis. 2017;14:E24. https://doi.org/10.5888/pcd14.160287 PMID: 28301314
7. Wu SH, Liu Z, Ho SC. Metabolic syndrome and all-cause mortality: a meta-analysis of prospective cohort studies. Eur J Epidemiol. 2010;25(6):375–84. https://doi.org/10.1007/s10654-010-9459-z PMID: 20425137
8. Wens I, Dalgas U, Stenager E, Eijnde BO. Risk factors related to cardiovascular diseases and the metabolic syndrome in multiple sclerosis - a systematic review. Mult Scler. 2013;19(12):1556–64. https://doi.org/10.1177/1352458513504252 PMID: 24048545
9. Sun J, Qiao Y, Zhao M. Magnussen CG, Xi B. Global, regional, and national burden of cardiovascular diseases in youths and young adults aged 15–39 years in 204 countries/territories, 1990–2019: a systematic analysis of Global Burden of Disease Study 2019. BMC Med. 2023;21:222. https://doi.org/10.1186/s12916-023-02925-4 PMID: 37365627
10. Tong Z, Xie Y, Li K, Yuan R, Zhang L. The global burden and risk factors of cardiovascular diseases in adolescent and young adults, 1990-2019. BMC Public Health. 2024;24(1):1017. https://doi.org/10.1186/s12889-024-18445-6 PMID: 38609901
11. Danso KA, Appah G, Akuaku RS, Karikari YS, Ansong AK, Edwin F, et al. Prevalence of Congenital Heart Disease in Children and Adolescents Under 18 in Africa: A Systematic Review and Meta-Analysis. World J Pediatr Congenit Heart Surg. 2024;16(3):357–67. https://doi.org/10.1177/21501351241299405 PMID: 39703055
12. Olatunbosun S, Kaufman J, Cooper R, Bella A. Hypertension in a black population: prevalence and biosocial determinants of high blood pressure in a group of urban Nigerians. J Hum Hypertens. 2000;14(4):249–57. https://doi.org/10.1038/sj.jhh.1000975
13. Adedapo AD, Fawole O, Bamgboye AE, Adedapo K, Demmisie K, Osinubi O. Morbidity and mortality patterns of medical admissions in a Nigerian secondary health care hospital. African Journal of Medicine and Medical Sciences. 2012;41:13–20.
14. Ogah OS, Okpechi I, Chukwuonye II, Akinyemi JO, Onwubere BJ, Falase AO, et al. Blood pressure, prevalence of hypertension and hypertension related complications in Nigerian Africans: A review. World J Cardiol. 2012;4(12):327–40. https://doi.org/10.4330/wjc.v4.i12.327 PMID: 23272273
15. Senbanjo IO, Oshikoya KA. Obesity and blood pressure levels of adolescents in Abeokuta, Nigeria. Cardiovasc J Afr. 2012;23(5):260–4. https://doi.org/10.5830/CVJA-2011-037 PMID: 22038484

16. Tucker KL, Buranapin S. Nutrition and aging in developing countries. J Nutr. 2001;131(9):2417S-23S. https://doi.org/10.1093/jn/131.9.2417S PMID: 11533287
17. Misra A, Singhal N, Khurana L. Obesity, the metabolic syndrome, and type 2 diabetes in developing countries: role of dietary fats and oils. J Am Coll Nutr. 2010;29(3 Suppl):289S-301S. https://doi.org/10.1080/07315724.2010.10719844 PMID: 20823489
18. M. Afolabi B. Food Consumption Habits Among Black African Adolescents With Metabolic Syndrome In Lagos State, Nigeria. AJMCRR. 2024;03(05):01–20. https://doi.org/10.58372/2835-6276.1168
19. Odey F, Udoh A, Bassey I. Lipid profile of apparently healthy adolescents in Calabar, Nigeria. Cent Afr J Med. 2010;53:11–8. https://doi.org/10.4314/cajm.v53i1-4.62605
20. Yar'adua AI, Shuaibu L, Madaha M. A comparative analysis of lipid profile among rural and urban school going male adolescents in Katsina State, Nigeria. International Journal of Biochemistry Research & Review. 2015;5(4):250–8. https://doi.org/10.9734/IJBcRR/2015.029
21. Onyiriuka AN, Iduoriyekemwen NJ, Sadoh WE. Prevalence and pattern of dyslipidaemia among Nigerian adolescents living in Benin City: A school-based cross-sectional study. Sri Lanka J Child Health. 2021;50(3):385. https://doi.org/10.4038/sljch.v50i3.9685
22. Shay CM, Ning H, Daniels SR, Rooks CR, Gidding SS, Lloyd-Jones DM. Status of cardiovascular health in US adolescents: prevalence estimates from the National Health and Nutrition Examination Surveys (NHANES) 2005-2010. Circulation. 2013;127(13):1369–76. https://doi.org/10.1161/CIRCULATIONAHA.113.001559 PMID: 23547177
23. Klotz O, Manning MF. Fatty streaks in the intima of arteries. J Pathol Bacteriol. 1911;16:211–20.
24. Strong JP, Malcom GT, McMahan CA, Tracy RE, Newman WP 3rd, Herderick EE, et al. Prevalence and extent of atherosclerosis in adolescents and young adults: implications for prevention from the Pathobiological Determinants of Atherosclerosis in Youth Study. JAMA. 1999;281(8):727–35. https://doi.org/10.1001/jama.281.8.727 PMID: 10052443
25. Gaziano JM, Hennekens CH, O'Donnell CJ, Breslow JL, Buring JE. Fasting triglycerides, high-density lipoprotein, and risk of myocardial infarction. Circulation. 1997;96(8):2520–5. https://doi.org/10.1161/01.cir.96.8.2520 PMID: 9355888
26. Kannel WB, Vasan RS, Keyes MJ, Sullivan LM, Robins SJ. Usefulness of the triglyceride-high-density lipoprotein versus the cholesterol-high-density lipoprotein ratio for predicting insulin resistance and cardiometabolic risk (from the Framingham Offspring Cohort). Am J Cardiol. 2008;101(4):497–501. https://doi.org/10.1016/j.amjcard.2007.09.109 PMID: 18312765
27. Bertoluci MC, Quadros AS, Sarmento-Leite R, Schaan BD. Insulin resistance and triglyceride/HDLc index are associated with coronary artery disease. Diabetol Metab Syndr. 2010;2:11. https://doi.org/10.1186/1758-5996-2-11 PMID: 20181078
28. Rezapour M, Shahesmaeili A, Hossinzadeh A, Zahedi R, Najafipour H, Gozashti MH. Comparison of Lipid Ratios to Identify Metabolic Syndrome. Arch Iran Med. 2018;21(12):572–7. PMID: 30634854
29. Di Bonito P, Valerio G, Grugni G, Licenziati MR, Maffeis C, Manco M, et al. Comparison of non-HDL-cholesterol versus triglycerides-to-HDL-cholesterol ratio in relation to cardiometabolic risk factors and preclinical organ damage in overweight/obese children: the CARITALY study. Nutr Metab Cardiovasc Dis. 2015;25(5):489–94. https://doi.org/10.1016/j.numecd.2015.01.012 PMID: 25813687
30. Quijada Z, Paoli M, Zerpa Y, Camacho N, Cichetti R, Villarroel V, et al. The triglyceride/HDL-cholesterol ratio as a marker of cardiovascular risk in obese children; association with traditional and emergent risk factors. Pediatr Diabete. 2023.
31. Di Bonito P, Moio N, Scilla C, Cavuto L, Sibilio G, Sanguigno E, et al. Usefulness of the high triglyceride-to-HDL cholesterol ratio to identify cardiometabolic risk factors and preclinical signs of organ damage in outpatient children. Diabetes Care. 2012;35(1):158–62. https://doi.org/10.2337/dc11-1456 PMID: 22040842
32. Murguía-Romero M, Jiménez-Flores JR, Sigrist-Flores SC, Espinoza-Camacho MA, Jiménez-Morales M, Piña E, et al. Plasma triglyceride/HDL-cholesterol ratio, insulin resistance, and cardiometabolic risk in young adults. J Lipid Res. 2013;54(10):2795–9. https://doi.org/10.1194/jlr.M040584 PMID: 23863983
33. Salazar MR, Carbajal HA, Espeche WG, Leiva Sisnieguez CE, Balbín E, Dulbecco CA, et al. Relation among the plasma triglyceride/high-density lipoprotein cholesterol concentration ratio, insulin resistance, and associated cardio-metabolic risk factors in men and women. Am J Cardiol. 2012;109(12):1749–53. https://doi.org/10.1016/j.amjcard.2012.02.016 PMID: 22449634
34. Awujoola AO, Torga AP, Valencia JE, Alemneh H, Prince OP, Kant SP. Utility of the TG/HDL-C Ratio in Estimating Pediatric Cardiometabolic Risk in a Community Hospital. Children (Basel). 2024;11(11):1277. https://doi.org/10.3390/children11111277 PMID: 39594852
35. Kosmas CE, Rodriguez Polanco S, Bousvarou MD, Papakonstantinou EJ, Peña Genao E, Guzman E, et al. The Triglyceride/High-Density Lipoprotein Cholesterol (TG/HDL-C) Ratio as a Risk Marker for Metabolic Syndrome and Cardiovascular Disease. Diagnostics (Basel). 2023;13(5):929. https://doi.org/10.3390/diagnostics13050929 PMID: 36900073
36. Lagos State Ministry of Education Directory. 2011. https://lagosstate.gov.ng/services/education
37. WHO. Adolescent health. n.d. [cited 2022 October 22]. https://www.who.int/southeastasia/health-topics/adolescenthealth
38. Holdbrooke SJ, Afolabi BM, Onabanjo OO, Salau IO, Lucas OT. Linking anthropometric indices with components of metabolic syndrome among Nigerian adolescents. Academia Medicine. 2025;2(1). https://doi.org/10.20935/acadmed7492
39. National Cholesterol Education Program (NCEP). Expert Panel on Blood Cholesterol Levels in Children and Adolescents: highlight of the reports of the Expert Panel. Pediatrics. 1992;89(3):495–501. https://doi.org/10.1542/peds.89.3.495

40. Lande MB, Batisky DL. New American Academy of Pediatrics Hypertension Guideline. Hypertension. 2019;73(1):31–2. https://doi.org/10.1161/HYPERTENSIONAHA.118.11819 PMID: 30571567
41. Afolabi BM, Holdbrooke SJ. Obesity, Dyslipidemia and other Risks Factors for Metabolic Syndrome among Indigenous Black African Secondary School Students in Lagos, Nigeria. Qeios Ltd. 2023. https://doi.org/10.32388/s522vg
42. Li H-Y, Chen B-D, Ma Y-T, Yang Y-N, Ma X, Liu F, et al. Optimal cutoff of the triglyceride to high-density lipoprotein cholesterol ratio to detect cardiovascular risk factors among Han adults in Xinjiang. J Health Popul Nutr. 2016;35(1):30. https://doi.org/10.1186/s41043-016-0067-8 PMID: 27586369
43. Liang H, Chen X, Chen Q, Wang Y, Wu X, Li Y, et al. The metabolic syndrome among postmenopausal women in rural Canton: prevalence, associated factors, and the optimal obesity and atherogenic indices. PLoS One. 2013;8(9):e74121. https://doi.org/10.1371/journal.pone.0074121 PMID: 24040183
44. Zhang L, Chen S, Deng A, Liu X, Liang Y, Shao X, et al. Association between lipid ratios and insulin resistance in a Chinese population. PLoS One. 2015;10. https://doi.org/10.1371/journal.pone.0116110
45. Arthur FKN, Adu-Frimpong M, Osei-Yeboah J, Mensah FO, Owusu L. Prediction of metabolic syndrome among postmenopausal Ghanaian women using obesity and atherogenic markers. Lipids Health Dis. 2012;11:101. https://doi.org/10.1186/1476-511X-11-101 PMID: 22883105
46. Pan S, Yu Z-X, Ma Y-T, Liu F, Yang Y-N, Ma X, et al. Appropriate body mass index and waist circumference cutoffs for categorization of overweight and central adiposity among Uighur adults in Xinjiang. PLoS One. 2013;8(11):e80185. https://doi.org/10.1371/journal.pone.0080185 PMID: 24244645
47. Murguía-Romero M, Jiménez-Flores JR, Sigrist-Flores SC, Espinoza-Camacho MA, Jiménez-Morales M, Piña E, et al. Plasma triglyceride/HDL-cholesterol ratio, insulin resistance, and cardiometabolic risk in young adults. J Lipid Res. 2013;54(10):2795–9. https://doi.org/10.1194/jlr.M040584 PMID: 23863983
48. Bovet P, Faeh D, Gabriel A, Tappy L. The prediction of insulin resistance with serum triglyceride and high-density lipoprotein cholesterol levels in an East African population. Arch Intern Med. 2006;166(11):1236–7. https://doi.org/10.1001/archinte.166.11.1236-b PMID: 16772257
49. Moriyama K, Urata N, Masuda Y, Oda K, Okuno C, Yamada C, et al. Usefulness of Triglyceride to High-Density Lipoprotein Ratio and Alanine Aminotransferase for Predicting Insulin Resistance and Metabolic Syndrome in the Japanese Population. Metab Syndr Relat Disord. 2021;19(4):225–32. https://doi.org/10.1089/met.2020.0121 PMID: 33577395
50. Sumner AE, Finley KB, Genovese DJ, Criqui MH, Boston RC. Fasting triglyceride and the triglyceride-HDL cholesterol ratio are not markers of insulin resistance in African Americans. Arch Intern Med. 2005;165(12):1395–400. https://doi.org/10.1001/archinte.165.12.1395 PMID: 15983289
51. Baneu P, Văcărescu C, Drăgan S-R, Cirin L, Lazăr-Höcher A-I, Cozgarea A, et al. The Triglyceride/HDL Ratio as a Surrogate Biomarker for Insulin Resistance. Biomedicines. 2024;12(7):1493. https://doi.org/10.3390/biomedicines12071493 PMID: 39062066
52. Li C, Ford ES, Meng Y-X, Mokdad AH, Reaven GM. Does the association of the triglyceride to high-density lipoprotein cholesterol ratio with fasting serum insulin differ by race/ethnicity?. Cardiovasc Diabetol. 2008;7:4. https://doi.org/10.1186/1475-2840-7-4 PMID: 18307789
53. Dharuni R, Maruthi Prasad BV, Vishwanath HL, Harish R. Evaluation of triglyceride: HDL-C ratio and Non-HDL-C as harbingers of increased cardiovascular risk in metabolic syndrome. Int J Res Med Sci. 2016;4(9):4050–4. https://doi.org/10.18203/2320-6012.ijrms20162932
54. Bashir B, Schofield J, Downie P, France M, Ashcroft DM, Wright AK, et al. Beyond LDL-C: unravelling the residual atherosclerotic cardiovascular disease risk landscape-focus on hypertriglyceridaemia. Front Cardiovasc Med. 2024;11:1389106. https://doi.org/10.3389/fcvm.2024.1389106 PMID: 39171323
55. Bashir B, Ho JH, Downie P, Hamilton P, Ferns G, Datta D, et al. Severe Hypertriglyceridaemia and Chylomicronaemia Syndrome—Causes, Clinical Presentation, and Therapeutic Options. Metabolites. 2023;13(5):621. https://doi.org/10.3390/metabo13050621
56. Hidalgo NJ, Pando E, Alberti P, Vidal L, Mata R, Fernandez N, et al. Elevated Serum Triglyceride Levels in Acute Pancreatitis: A Parameter to be Measured and Considered Early. World j surg. 2022;46(7):1758–67. https://doi.org/10.1007/s00268-022-06533-w
57. da Luz PL, Favarato D, Faria-Neto JR Jr, Lemos P, Chagas ACP. High ratio of triglycerides to HDL-cholesterol predicts extensive coronary disease. Clinics (Sao Paulo). 2008;63(4):427–32. https://doi.org/10.1590/s1807-59322008000400003 PMID: 18719750
58. Shuo P, Yitong M. Best single predictor of metabolic syndrome via comparing the predicting ability of various anthropometric and atherogenic parameters among Uighur population in Xinjiang. J Am Coll Cardiol. 2014;64(16_S).
59. Jeppesen J, Hein HO, Suadicani P, Gyntelberg F. Triglyceride concentration and ischemic heart disease: an eight-year follow-up in the Copenhagen Male Study. Circulation. 1998;97(11):1029–36. https://doi.org/10.1161/01.cir.97.11.1029 PMID: 9531248
60. Edwards KL, Mahaney MC, Motulsky AG, Austin MA. Pleiotropic genetic effects on LDL size, plasma triglyceride, and HDL cholesterol in families. Arterioscler Thromb Vasc Biol. 1999;19(10):2456–64. https://doi.org/10.1161/01.atv.19.10.2456 PMID: 10521376
61. Brinton EA, Eisenberg S, Breslow JL. Increased apo A-I and apo A-II fractional catabolic rate in patients with low high density lipoprotein-cholesterol levels with or without hypertriglyceridemia. J Clin Invest. 1991;87(2):536–44. https://doi.org/10.1172/JCI115028 PMID: 1899429
